# Presynaptic Mechanisms and KCNQ Potassium Channels Modulate Opioid Depression of Respiratory Drive

**DOI:** 10.3389/fphys.2019.01407

**Published:** 2019-11-22

**Authors:** Aguan D. Wei, Jan-Marino Ramirez

**Affiliations:** ^1^Seattle Children’s Research Institute, Center for Integrative Brain Research, Seattle, WA, United States; ^2^Department of Neurological Surgery, University of Washington School of Medicine, Seattle, WA, United States

**Keywords:** opioid, respiratory depression, presynaptic, KCNQ, preBötC

## Abstract

Opioid-induced respiratory depression (OIRD) is the major cause of death associated with opioid analgesics and drugs of abuse, but the underlying cellular and molecular mechanisms remain poorly understood. We investigated opioid action *in vivo* in unanesthetized mice and in *in vitro* medullary slices containing the preBötzinger Complex (preBötC), a locus critical for breathing and inspiratory rhythm generation. Although hypothesized as a primary mechanism, we found that mu-opioid receptor (MOR1)-mediated GIRK activation contributed only modestly to OIRD. Instead, mEPSC recordings from genetically identified *Dbx1-*derived interneurons, essential for rhythmogenesis, revealed a prevalent presynaptic mode of action for OIRD. Consistent with MOR1-mediated suppression of presynaptic release as a major component of OIRD, *Cacna1a* KO slices lacking P/Q-type Ca^2+^ channels enhanced OIRD. Furthermore, OIRD was mimicked and reversed by KCNQ potassium channel activators and blockers, respectively. *In vivo* whole-body plethysmography combined with systemic delivery of GIRK- and KCNQ-specific potassium channel drugs largely recapitulated these *in vitro* results, and revealed state-dependent modulation of OIRD. We propose that respiratory failure from OIRD results from a general reduction of synaptic efficacy, leading to a state-dependent collapse of rhythmic network activity.

## Introduction

Respiratory depression is the primary cause of death associated with opioid-based analgesics and drugs of abuse. In 2015, the opioid epidemic claimed 33,091 deaths in the US, accounting for 16.3 deaths per 100,000 (Rudd et al., 2016). Despite extensive investigations, central mechanisms of opioid action on respiratory drive remain incompletely resolved (Shook et al., 1990; Dahan et al., 2009). Identifying cellular and molecular mechanisms underlying Opioid-Induced Respiratory Depression (OIRD) may provide insights for reversing OIRD and controlling *in vivo* variability.

The mammalian respiratory motor program appears to assemble from three rhythmogenic medullary microcircuits (Feldman et al., 2012; Anderson and Ramirez, 2017): The preBötzinger Complex (preBötC), responsible for inspiratory rhythm generation (Smith et al., 1991), the Retrotrapezoid/Parafacial nucleus (RTN/pFRG) (Onimaru et al., 2008; Thoby-Brisson et al., 2009; Huckstepp et al., 2016), critical for active expiration, and the Post-inspiratory Complex (PiCo), associated with post-inspiratory activity (Anderson et al., 2016). Only preBötC and PiCo are suppressible by opioids (Mellen et al., 2003; Anderson et al., 2016). Although opioid receptors are distributed widely in the brainstem, including at sites likely to contribute to respiratory depression such as the Kölliker-Fuse/Parabrachial nuclei (Erbs et al., 2015; Cerritelli et al., 2016), only the preBötC is essential for breathing and survival (Ramirez et al., 1998; Gray et al., 2001, 2010; Tan et al., 2008; although see Lalley et al., 2014). We thus focused on OIRD mechanisms in the preBötC, a site responsible for apneas when infused with opioids (Greer et al., 1995; Mellen et al., 2003; Montandon et al., 2011; Montandon and Horner, 2014a, b).

Opioid receptors comprise four G-protein coupled receptors (GPCRs) (mu-, delta-, kappa-, nociceptin/orphanin FQ), which signal through the canonical Gα_i/o_ intracellular signaling pathway. Mouse KO studies demonstrate that mu-opioid receptors (OPRM1, hereafter referred to as MOR1) are primarily responsible for adverse non-analgesic side-effects of OIRD and suppression of gastric motility (Sarton et al., 2001; Bohn and Raehal, 2006). Ion channels known to act downstream of activated MOR1 to suppress excitability include presynaptic voltage-gated calcium channels (N-, P/Q- and R-type), whose gating is suppressed by Gβ/γ released from activated GPCRs (Dunlap and Fischbach, 1978; Herlitze et al., 1996; Ikeda, 1996; Colecraft et al., 2000; Zamponi and Currie, 2012), and GIRK potassium channels directly activated by Gβ/γ binding (Logothetis et al., 1987; Reuveny et al., 1994; Krapivinsky et al., 1995; Whorton and MacKinnon, 2013). KCNQ (Kv7) potassium channels are another attractive class of effectors (Jentsch, 2000) possibly modulating OIRD, based on reports of up-modulation by somatostatin receptors in neurons (Moore et al., 1988; Qiu et al., 2008), and β-adrenergic receptors in smooth muscle (Sims et al., 1988). We identified a role for KCNQ channels in modulating OIRD, however acting independent of coupling by intracellular signaling from MOR1.

We combined pharmacological and genetic approaches in mice, employing both *in vitro* electrophysiological recordings from rhythmic preBötC slices, and *in vivo* plethysmography without anesthesia.

Unexpectedly, *in vitro* OIRD produced by bath-applied DAMGO (a MOR1-specific agonist), was not reversed by TertiapinQ, a GIRK-specific blocker, suggesting a minimal role for increased GIRK conductance resulting from MOR1 activation. In addition, application of ML297, a GIRK-specific activator failed to mimic DAMGO-mediated *in vitro* OIRD. By contrast, genetic removal of Ca_V_2.1 (P/Q-type calcium channel) in *Cacna1a* KO mice, sensitized *in vitro* preBötC rhythms to DAMGO depression, consistent with OIRD acting through MOR1-mediated inhibition of presynaptic calcium channels. We similarly identified KCNQ potassium channels as a novel modulator of OIRD, but independent of coupling to MOR1 signaling. KCNQ-specific blockers (Chromanol 293B, XE991) reversed, and activators (retigabine, ICA 69673) mimicked *in vitro* OIRD. To test for a presynaptic site of action, miniature excitatory post-synaptic currents (mEPSCs) were recorded in TTX from genetically identified *Dbx1*-expressing inspiratory interneurons (Bouvier et al., 2010). DAMGO suppressed mEPSC frequency significantly, and subsequent XE991 application restored mEPSC frequencies in ∼70% of these neurons. *In vivo* plethysmography corroborated these *in vitro* findings: systemic XE991 reversed morphine-induced OIRD in an apparent state-dependent fashion, whereas ML297 (GIRK1-specific activator) failed to suppress. Moreover, retigabine (KCNQ-specific activator) dramatically depressed respiratory frequencies. These results support a presynaptic site of modulatory action by KCNQ potassium channels, acting in concert with MOR1-activated suppression of voltage-gated calcium channels as the predominant mechanism underlying OIRD in the preBötC.

## Materials and Methods

### Animal Welfare

All animal procedures were approved by IACUC, Seattle Children’s Research Institute (SCRI).

### Animal Strains

Animals were maintained under a 12/12 light/dark cycle in the SCRI vivarium. Mouse strains used include:

(1) *Cacna1a* (Ca_V_2.1; P/Q-type) KO, C3H background (Jun et al., 1999), gift of Christopher Gomez (Univ. Chicago).
(2) *Dbx1*^*CreERT2*^, CD1 background (Hirata et al., 2009), gift of Christopher Del Negro (The College of William and Mary). This strain was out-crossed from CD1 to C57BL/6J background for animals in this study.
(3) *B6.Cg-Gt(ROSA)26Sor^*tm6(CAG–ZsGreen1)Hze*^/J* (Ai6) (Madisen et al., 2012), from JAX (Stock No. 007906).
(4) *B6.Cg-Gt(ROSA)26Sor^*tm14(CAG–tdTomato)Hze*^/J* (Ai14) (Madisen et al., 2012), from JAX (Stock No. 007914).
(5) B6.Cg-Gt(ROSA)26Sor^tm27.1(CAG–COP4*H134R/tdTomato)Hze^/J (Ai27) (Madisen et al., 2012), from JAX (Stock No. 012567).
(6) C3H, out-bred from the *Cacna1a* KO line used above.
(7) C57BL/6J, from JAX (Stock No. 000664).

For genotyping, genomic DNA was extracted from tail clippings by the DNAeasy Blood and Tissue Kit (Qiagen). Genotyping was performed by PCR with the following primer sets, resolved by electrophoresis on 0.5X TBE agarose gels (1.0–2.0%):

(1) For *Cacna1a* (Ca_V_2.1) KO, a single multiplexed PCR was performed for each animal with four primers targeting exon 4 of *Cacna1a*, deleted in the mutant line:
  5′-CGTTCCTTGCGCACGTGTGCT C-3′
  5′-GGGATCATCGCCTTCATGATTGACTTCAGGACGA CT-3′.
  5′-CTGACCCTAATCCAACTATTCAGCCATCCCGAGT CT-3′
  5′-ACAGAAGGAATTCTATGAGTTCAGTAACAGCCTG GGCTA-3′.
  This reaction generates products of 1500 bp for copies of the *Cacna1a* deletion and 930 bp for WT *Cacna1a*.
(2) For *Dbx1*^*CreERT2*^, a single multiplexed PCR was performed for each animal with four primers targeting the site of insertion of ERT2-modified Cre in the 3′UTR of *Dbx1*:
  5′-GCGGTCTGGCAGTAAAAACTATC-3′
  5′-GTGAAACAGCATTGCTGTCACTT-3′
  5′-GCCCGGGTAAACCGTCAGACTTC-3′
  5′-GCTCTTGTAGAAAAGACCCACGCTCC-3′
  This reaction generates products of 106 bp for copies of *Dbx1*^*Cre–ERT2*^, and 310 bp for WT *Dbx1*.
(3) For Ai6, Ai14, Ai27, a single multiplexed PCR was performed for each animal with three primers targeting the insertion site of the CAG-floxed-stop expression cassettes in the *Rosa26* locus:
  5′-CCTCGTGATCTGCAACTCCAGTCTTTC-3′
  5′-CAAGCAATAATAACCTGTAGTTTTGCTGCAT AA-3′
  5′-GGAACTCCATATATGGGCTATGAACTAATGA-3′
  This reaction generates products of 122 bp for copies of the transgenic cassette (Ai6, Ai14, Ai27), and 248 bp for WT *Rosa26*.

### Respiratory Slices

Transverse medullary respiratory slices (600 μM) containing the preBötC and hypoglossal nuclei (XII) were cut from neonatal mice (P7–13; mixed genders) on a Leica VT100S vibrotome (Leica Biosystems, Buffalo Grove, IL), as previously described (Koch et al., 2013). Transverse slices do not include PiCo (Anderson et al., 2016). Slices were assayed in oxygenated ACSF (118 NaCl, 3.0 KCl, 25 NaHCO_3,_ 1.0 NaH_2_PO_4_, 30 glucose, 1.0 MgCl_2_, 1.5 CaCl_2_, in mM; saturating 95% O_2_, 5% N_2_) at 30°, under rapid recirculating bath perfusion (∼13 mL/min). Two WT strains were used, C57BL6 and C3H. Individual slices were genotyped *post hoc* after recordings with DNA extracted from either tail clippings or from the recorded slice.

### Extracellular Recordings

Fictive inspiratory bursts were recorded as integrated multi-unit recordings from large bore glass pipettes filled with ACSF (∼0.1 MΩ) placed over the preBötC region, on the rostral face of respiratory slices. Slices were maintained under rapidly recirculating bath perfusion (13 mL/min) with oxygenated ACSF at 30°. Extracellular signals were acquired with an AC differential amplifier (Model 1700; A-M Systems, Sequim, WA), bandpass filtered between 0.1 and 10 KHz, and amplified x10K. Raw extracellular signals were acquired at 10 KHz with pClamp10.4. In parallel, extracellular signals were recorded after on-line processing through a custom analog signal integrator (Univ. Chicago Electronic Lab), which rectified and integrated transients using a τ = 60 ms. Output from this integrator is dependent upon both the frequency of clustered transients and the amplitude of individual events, and enhances the detection of high frequency multi-unit spike bursts (>17 Hz) under these conditions. ACSF was supplemented to 8 mM K^+^ over 30 min to evoke fictive inspiratory rhythms. Drugs were bath applied in 10 min intervals, and burst frequency measurements made during the last 2 min of drug application. All experiments employing sequential application of drugs were performed as a series on individual slices, then normalized to baseline frequencies for analysis. Bath exchange time was estimated to be 2–3 min, under our experimental conditions.

### Intracellular Recordings

Whole-cell voltage-clamp recordings were made from preBötC inspiratory neurons under visual control using video-enhanced Dodt-IR optics and fluorescence for either ZsGreen (Ai6) or tdTomato (Ai14) (Koch et al., 2013), on a Zeiss Axio Examiner.A1 microscope with a 40X water-immersion objective. Patch-clamp recordings were acquired with an Axopatch 1D amplifier and pClamp10.4 (Molecular Devices, Sunnyvale, CA, United States), digitized at 10 KHz, and filtered at 2 KHz. Patch-clamp electrodes were filled with low Cl^–^ internal solution (140 K^+^-gluconate, 1.0 CaCl_2_, 10 HEPES, 2.4 EGTA, 2.0 MgCl_2_, 2.0 Na_2_-ATP, 0.3 Na_2_-GTP; in mM), with resistances between 3.0 and 5.0 MΩ. Neurons were voltage-clamped at -60 mV for measurements of post-synaptic currents. Access resistances were typically < 0.6 MΩ, holding at -60 mV, with no series resistance compensation. Inspiratory neurons were identified by regular bursts of EPSCs with a frequency of ∼0.1–0.05 Hz, confirmed in some cases by simultaneous extracellular population recordings with a second extracellular electrode placed in the preBötC. To record mEPSCs, spontaneous presynaptic action potentials were blocked with 1.0 μM TTX; DAMGO (100 nM) was subsequently bath applied, followed by XE991 (20 μM). mEPSCs were analyzed with Clampfit10.4 (Molecular Devices, Sunnyvale, CA, United States) and Mini Analysis 6.0.3 (Synaptosoft, Decatur, GA, United States). Slices during visual patch-clamp recording were maintained in oxygenated ACSF at 30°, under continuous recirculation perfusion (∼3 mL/min).

### *In vivo* Whole-Body Plethysmography

Ventilatory function was assessed by whole-body plethysmography under unrestrained normoxic conditions, under a constant flow (5.8 psi) of standard air (Buxco Research System/DSI, St. Paul, MN, United States). Neonatal (P7–13) and adult (P25–60) animals of mixed genders were used. Recording sessions consisted of 10 min of baseline, followed by 30–60 min of recording following drug delivery. Drugs were delivered intraperotineally (IP) with 31 gauge insulin syringes using minimal volumes (<30 μL for neonates; <250 μL for adults). Drug series and dosages included: (1) ML297 (50 mg/kg), (2) Retigabine (10 mg/kg), (3) Morphine (10 mg/kg for neonates; 150 mg/kg for adults) followed by XE991 (3 mg/kg) (4) DMSO vehicle control. All experiments employing sequential application of drugs were performed as a series on individual animals, then normalized to baseline frequencies for analysis. Drugs were dissolved in 100% DMSO due to poor solubility in saline, to limit injection volumes. Plethysmography signals were acquired as differential pressure signals, relative to equally pressurized control chambers, using pClamp 10.4 and analyzed with Clampfit10 (Molecular Devices, Sunnyvale, CA, United States). Calibration pressure pulses were generated with a manual pipettor (P20, Eppendorf AG, Enfield, CT, United States).

For analysis of respiratory frequency, a large number of breaths were curated (500–1000 events) per animal for each measurement, manually excluding large amplitude irregular movement artifacts and rapid high amplitude episodes (>5 Hz) representing active whisking/sniffing (Moore et al., 2013). The distributions of these events were fitted to single Guassian distributions in Origin 8 (OriginLabs, Northhampton, MA, United States), and further statistically analyzed and plotted in Prism 5.04 (GraphPad Software, San Diego, CA, United States).

### RT-PCR of preBötC Islands

RT-PCR followed standard procedures. Briefly, total RNA was extracted from preBötC “islands,” micro-dissected and pooled from freshly cut preBötC slices with RNAzol RT (Molecular Research Center, Inc, Cincinnati, OH). First-strand synthesis of single-stranded cDNA (sscDNA) was generated with 1.0–5.0 μg total RNA, primed with random hexamers, and reverse transcriptase following the vendor’s protocol (SuperScript IV, Thermo Fisher Scientific). Control reactions without reverse transcriptase were generated in parallel, as a control for residual genomic DNA. 2.0 μL (1:100 dilution) of sscDNA was used for each RT-PCR with gene-specific primer sets and 35–40 cycles of amplification, using GoTaq Hot Start Master Mix (Promega, Madison, WI, United States). See Table 1 for RT-PCR primer sequences.

**TABLE 1:** Primer sequences for RT-PCR.

	**Gene**	**Strand**	**Primer (5′ > 3′)**	**Product size (bp)**
1	Mouse Kcnq1	F	GCTACGCAGATGCTCTGTGGTGG	70
		R	TGAGGTACCTTATCCCCGTAGCCAA	
2	Mouse Kcnq2	F	TGACTGCCTGGTACATTGGC	264
		R	CTCTTGGACTTTCAGGGCAAA	
3	Mouse Kcnq3	F	CAGCACCGTCAGAAGCACTTTGA	117
		R	TCTCCAGGTTGCCACCAGATCC	
4	Mouse Kcnq4	F	ATATAATCCGTGTCTGGTCGGC	123
		R	GACGTAGCAAAGATGTTGCCT	
5	Mouse Kcnq5	F	CGCACTCCTTGGCATTTCTTTCTTT	89
		R	TCTGGCGGTGCTGCTCCTGTA	
6	Mouse Oprm1	F	CTATCGTGTGTGTAGTGGGCCTCTTTGG	114
		R	CTGCCAGAGCAAGGTTGAAAATGTAGAT	
7	Mouse Girk1 (Kcnj3)	F	CTATCGTGTGTGTAGTGGGCCTCTTTGG	102
		R	CTGCCAGAGCAAGGTTGAAAATGTAGAT	
8	Mouse Girk2 (Kcnj6)	F	ATTGATTATTAGCCATGAAATTAACCAAAGAGT	174
		R	TAACCCCACAAGATCTCACTGGTGATGTA	
9	Mouse Girk3 (Kcnj9)	F	CATTCTCGAGGGCATGGTGGAG	185
		R	GAGGTCGGTCTGGTGCAAAG	
10	Mouse Girk4 (Kcnj5)	F	AAAACCTTAGCGGCTTTGTATCT	152
		R	AAGGCATTAACAATCGAGCCC	
11	Mouse Sstr2	F	CGCATGGTGTCCATCGTAGT	246
		R	GGATTGTGAATTGTCTGCCTTGA	
12	Mouse Tacr1 (Nk1r)	F	CTCCACCAACACTTCTGAGTC	221
		R	TCACCACTGTATTGAATGCAGC	
13	Mouse Grpr1	F	TTGTTCCCACCTGAACTTGGA	169
		R	CGTGATGTTGCCAATAAGACCTA	
14	Mouse Sst	F	CAGCGGGCATGGTCACTATC	250
		R	CCGTCCACGCTAAGCACTG	
15	Mouse Tac1 (Nk1)	F	CAGTCACCAACTCAGTCCTGC	110
		R	CACAACGATCTCGAAGTCCCC	

### Heterologous Expression Studies in *Xenopus* Oocytes

*Xenopus* oocyte expression followed standard procedures. Full-length cDNAs were subcloned into plasmids (pOX or pcDNA3hnk) (Wei et al., 1990) and *in vitro* 5′ capped cRNAs transcribed with T3 RNA polymerase (mMessage mMachine, Ambion, Austin, TX). Oocytes were purchased commercially (Ecocyte, Austin, TX, United States) and injected with combinations of cRNAs (∼50 nL) diluted to ∼0.3–1.0 μg/μL, and incubated 4–7 days at 18° prior to two-electrode voltage-clamp recordings. cDNA clones were generously provided by the following investigators: rMOR1_GFP and GIRK1(F137S) (C. Chavkin, University of Washington), rKCNQ3 (D. McKinnon, SUNY-Stony Brook), and hKCNQ5 (K. Steinmeyer, Sanofi-Adventis, Germany). A full-length hKCNQ4 clone was assembled using a partial cDNA (NITE, Chiba, Japan) with additional 5′-biased cDNAs derived by RT-PCR from human tissue.

Two-electrode voltage-clamp recordings were made on a two-electrode voltage-clamp workstation (TEV-700/OC725C, Warner Instruments, Hamden, CT), as previously described (Wei et al., 1990). Recordings were acquired with pClamp10.4 (Molecular Devices, Sunnyvale, CA, United States).

### Drugs and Software

The following vendors provided DAMGO ([D-Ala^2^, NMe-Phe^4^, Gly-ol^5^]-enkephalin), XE991, Chromanol 293B ((-)-[3R,4S]-Chromanol 293B), retigabine, ICA 69673, TertiapinQ, and ML297: Tocris Biosciences/R&D Systems (Minneapolis, MN), Cayman Chemicals (Ann Arbor, MI), Alomone Labs (Jerusalem, Israel), Sigma-Aldrich/Millipore-Sigma (St. Louis, MO, United States). ML297 was also provided by C. David Weaver (Vanderbilt University). Morphine was purchased from Patterson Veterinary Supply (Greeley, CO, United States). Drug concentrations used were guided from the available literature (ion channel blockers and activators, morphine and DAMGO), or determined experimentally in this study in those cases where no pre-established data was available (see Table 2).

**TABLE 2:** Pharmacological properties of drugs and dosages used.

	**EC50**	**IC50**	**References**
**DAMGO**			
OPRM1	45 nM		Alt et al., 2002
**ICA 69673**			
KCNQ2/3	0.7 μM		Padilla et al., 2009; Wang et al., 2017
**Retigabine**			
KCNQ2/3	0.4–1.6 μM		Wuttke et al., 2005; Gunthorpe et al., 2012; Kim et al., 2015
KCNQ5/3	1.4 μM		Gunthorpe et al., 2012
KCNQ2	2.5 μM		Gunthorpe et al., 2012
KCNQ3	0.6 μM		Gunthorpe et al., 2012
KCNQ4	5.2 μM		Gunthorpe et al., 2012
KCNQ5	6.4 μM		Gunthorpe et al., 2012
**XE991**			
KCNQ2/3		0.6–1.0 μM	Wang et al., 1998, 2000
KCNQ1		0.75 μM	Wang et al., 2000
KCNQ4		5.5 μM	Schroder et al., 2001; Sogaard et al., 2001
KCNQ5		∼50 μM	Lerche et al., 2000; Schroeder et al., 2000
**Chromanol 293B**			
KCNQ1/KCNE1		∼11 μM	Lerche et al., 2000, 2007
KCNQ2/3		>500 μM	Lerche et al., 2007
KCNQ3		>500 μM	Lerche et al., 2007
KCNQ4		>500 μM	Lerche et al., 2007
KCNQ5		∼100 μM	Lerche et al., 2007
**ML297**			
GIRK1/2	0.16 μM		Kaufmann et al., 2013
GIRK1/3	0.91 μM		Kaufmann et al., 2013
GIRK1/4	0.89 μM		Kaufmann et al., 2013
**TertiapinQ**			
GIRK1/2		5.4 nM	Jin et al., 1999; Kubo et al., 2005
GIRK1/4		15 nM	Jin et al., 1999; Kubo et al., 2005
GIRK2		7 nM	Jin et al., 1999; Kubo et al., 2005; Li D. et al., 2016
GIRK4		10 nM	Jin et al., 1999; Kubo et al., 2005

***In vivo* plethysmography**	**Dosage (neonates)**	**Dosage (adults)**	**References**

Morphine Sulfate	10 mg/kg	150 mg/kg	Jacquet et al., 1976; Koek et al., 2012; this study
ML297	50 mg/kg	50 mg/kg	Kaufmann et al., 2013; Wydeven et al., 2014
Retigabine	10 mg/kg	10 mg/kg	Li et al., 2013; Ihara et al., 2016
XE991	3.0 mg/kg	3.0 mg/kg	This study

Data were analyzed in Clampfit10 (Molecular Devices, Sunnyvale, CA, United States), Mini Analysis 6.0.3 (Synaptosoft, Decatur, GA, United States), Origin8 (OriginLabs, Northampton, MA, United States), and Prism 5.04 (GraphPad Software, San Diego, CA, United States). DNA analysis and primer design were performed with DNASTAR/Lasergene (DNASTAR, Madison, WI, United States).

### Statistical Analysis

Datasets were tested for normality by D’Agostino and Pearson omnibus tests. Normal datasets were tested for statistical significance by unpaired *t*-tests (unless otherwise noted) assuming equal variance, with *p*-values determined by two-tails at the 95% confidence level. Non-parametric datasets were tested for statistical significance by Mann–Whitney tests with *p*-values determined by two-tails at the 95% confidence level. Miniature excitatory post-synaptic current (mEPSC) inter-event interval datasets were plotted as cumulative fractional distributions and tested for statistical significances by paired Wilcox-Signed Rank comparisons (modified Kolmogorov–Smirnov test), with *p*-values determined at the 95% confidence level (OriginLabs, Northampton, MA, United States).

Normal datasets are plotted as mean with standard error (SE). Non-parametric datasets are plotted as median with interquartile range (IQR). Statistical calculations were performed using Prism 5.04 (GraphPad Software, San Diego, CA, United States) and Origin8 (OriginLabs, Northampton, MA, United States).

## Results

### DAMGO Suppresses *in vitro* Rhythmic Inspiratory Bursts in prebötC Slices

**FIGURE 1:**
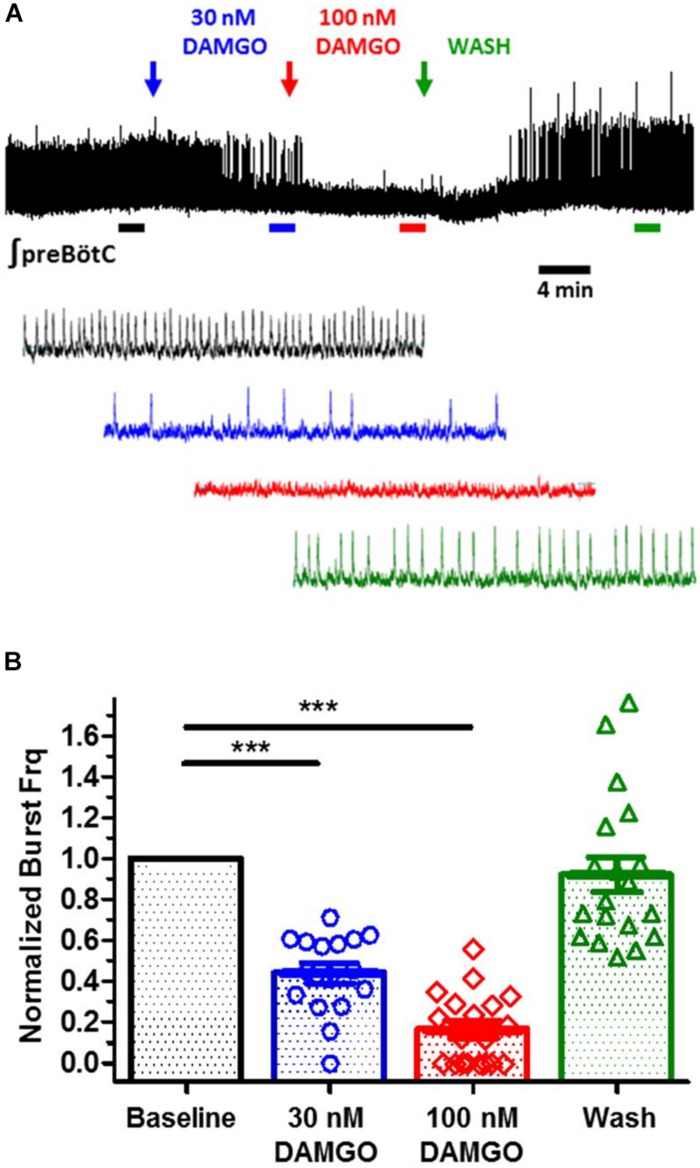
DAMGO, a mu-opioid receptor specific agonist, suppresses fictive inspiratory bursts recorded from *in vitro* preBötC medullary slices. **(A)** Integrated extracellular bursts recorded from an isolated preBötC, in response to bath application of increasing titers of DAMGO (30, 100 nM), followed by wash. Two minute segments shown at expanded time scale for baseline (black), 30 nM DAMGO (blue), 100 nM DAMGO (red), and wash (green). **(B)** Summary of burst frequency suppression by DAMGO, yielding an IC_50_ of ∼30 nM (*N* = 19 slices; ^∗∗∗^*p* < 0.0001; paired *t*-test). Wash restores burst frequency. Mean and SE plotted, with individual replicant values.

Rhythmic extracellular inspiratory bursts were reliably recorded from transverse slices containing the preBötC derived from neonatal C57BL/6J mice (P7–P13) in 8 mM extracellular K^+^, using blunt glass electrodes placed over the preBötC region. Inspiratory bursts were recognized as large amplitude integrated signals with a frequency of ∼0.02–0.3 Hz, confirmed in some cases by observing synchronous bilateral bursts from a second electrode placed over the contralateral preBötC. DAMGO ([D-Ala^2^, N-MePhe^4^, Gly-ol]-enkephalin), a MOR1-specific agonist, was bath applied to mimic the effect of central opioid action. Burst frequency was monitored as a highly sensitive measure of the integrity of the respiratory network, based on previous studies (Hayes et al., 2012; Wang et al., 2014; Guerrier et al., 2015; Song et al., 2015). Integrated burst amplitudes were largely unaffected by DAMGO, and provided a reliable means to monitor burst frequency, as an index for *in vitro* respiratory depression. Bath application of 30 nM DAMGO depressed *in vitro* inspiratory burst frequencies with an IC_50_ of ∼30 nM, consistent with the EC_50_ (45 nM) of DAMGO activation of MOR1 measured by [^35^S]-GTPγS accumulation (Alt et al., 2002). Inspiratory burst frequencies were suppressed to 10% of initial frequencies by 100 nM DAMGO, and restored by washout (Figure 1). Transverse slices thus provided an effective *in vitro* model system to study opioid action on the preBötC inspiratory network (Greer et al., 1995; Mellen et al., 2003; Ballanyi et al., 2009; Lorier et al., 2010).

### Activators of KCNQ Potassium Channels Depress Inspiratory Rhythmic Activity *in vitro*, Mimicking the Respiratory Effect of DAMGO

**FIGURE 2:**
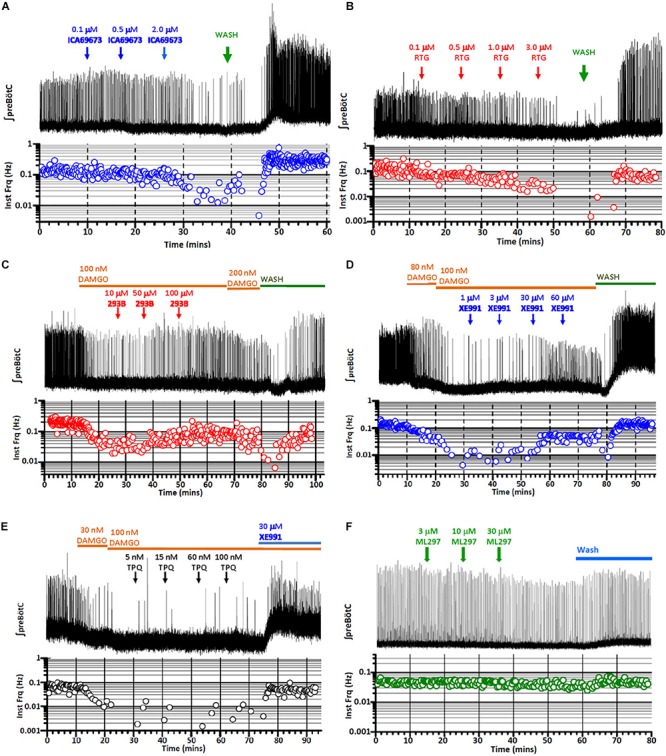
Exemplary records of fictive inspiratory bursts recorded from *in vitro* preBötC slices in response to increasing titers of activators or blockers of KCNQ and GIRK potassium channels, mimicking or reversing opioid-induced respiratory depression (OIRD). Integrated recording (top), instantaneous burst frequency (below). **(A)** Inspiratory bursts as a function of increasing titers of ICA 69673, a KCNQ activator (0.1, 0.5, 2.0, in mM), mimicking OIRD. **(B)** Inspiratory bursts as a function of increasing titers of retigabine (RTG), an FDA-approved KCNQ activator (0.1, 0.5, 1.0, 3.0, in mM), mimicking OIRD. **(C)** Rescue of DAMGO-induced OIRD (100 nM) with increasing titers of Chromanol 293B (293B), a KCNQ blocker (10, 50, 100, in mM). **(D)** Rescue of DAMGO-induced OIRD (100 nM) with increasing titers of XE991, a KCNQ blocker (1, 3, 30, 60, in mM). **(E)** Failure to rescue DAMGO-induced OIRD (100 nM) with increasing titers of TertiapinQ (TPQ), a GIRK blocker (5, 15, 60, 100, in nM). **(F)** Failure to mimic OIRD by increasing titers of ML297, a GIRK activator (3, 10, 30, in mM). Transient apneas observed at the beginning of washes **(A–D)** are an artifact of temperature drop or oxygen desaturation from perfusion exchanges.

**FIGURE 3:**
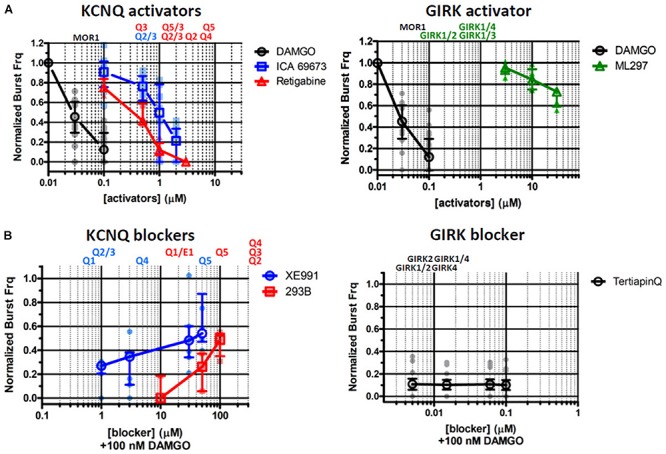
Pharmacological interrogations of *in vitro* preBötC slices suggests a modulatory role for KCNQ potassium channels in opioid-induced respiratory depression (OIRD), and a minor role for GIRK potassium channels. (**A**, left) Dose-response curves of inspiratory burst frequencies in response to increasing titers of DAMGO, and two activators of KCNQ potassium channels (ICA 69673, Retigabine) which act on different structural domains of the channel subunit. Both activators inhibit burst frequencies with an IC_50_ = ∼0.7–1.0 μM, comparable to their EC_50_ for activation of KCNQ channels. (**A**, right) Dose-response curves of inspiratory burst frequencies in response to increasing titers of ML297, a GIRK1 subunit specific activator. Modest depression of burst frequencies, at concentrations 10–100-fold higher than the EC_50_s (0.16–0.9 μM) for GIRK1 containing heteromeric channels. (**B**, left) Dose-response curves of inspiratory burst frequency in response to increasing titers of the KCNQ blockers XE991 and Chromanol 293B (293B), applied in the presence of DAMGO (100 nM) to suppress respiratory rhythms. (**B**, right) Dose-response curves of inspiratory burst frequency in response to increasing titers of TertiapinQ (TPQ), a GIRK-specific blocker, applied in the presence of DAMGO (100 nM). No recovery of respiratory burst frequency was observed at the highest concentration of TPQ (100 nM). By contrast, both KCNQ blockers partially rescued respiratory rhythms suppressed by DAMGO, at relatively high concentrations (20–100 μM). Shown above plots are the EC_50_s and IC_50_s of each compound for specific molecular species of homo- and hetero-tetrameric GIRK and KCNQ channels reported in the literature (see text and Table 2 for references). Mean and SE plotted for DAMGO and TertiapinQ; median and IQR plotted for all other datasets, along with individual replicant values: DAMGO (*N* = 35), ICA 69673 (*N* = 14), Retigabine (*N* = 6), ML297 (*N* = 6), XE991 (*N* = 7), Chromanol 293B (*N* = 5), TertiapinQ (*N* = 9).

KCNQ (Kv7) potassium channels are highly conserved voltage-gated potassium channels characterized by partial activation at subthreshold membrane potentials, exceptionally slow gating kinetics, and promiscuous coupling to diverse GPCRs (Brown and Adams, 1980; Wang et al., 1998; Jentsch, 2000; Wei et al., 2005; Sun and MacKinnon, 2017). These features make this class of potassium channels particularly well-suited to regulating basal membrane excitability, which in turn may critically influence neuronal network dynamics (Brown and Passmore, 2009; Honigsperger et al., 2015). We took advantage of two pharmacological activators specific for KCNQ potassium channels, ICA 69673 and retigabine to test the involvement of this class of potassium channels on preBötC inspiratory rhythms. These openers operate on different structural domains of KCNQ subunits, lending an additional test for specificity. ICA 69673 binds to residues in S3 in the voltage sensor domain of KCNQ subunits (Padilla et al., 2009; Wang et al., 2017), whereas retigabine binds to a hydrophobic pocket near the cytosolic base of the ion conduction pathway formed by S5 and S6 residues, including a critically conserved S5 tryptophan (KCNQ3 W265) (Schenzer et al., 2005; Wuttke et al., 2005; Lange et al., 2009; Kim et al., 2015). Both activators potently suppressed *in vitro* inspiratory burst frequency with IC_50_s (∼1.0 μM for ICA 69673; ∼0.5 μM for retigabine) comparable to their EC_50_s for activation of KCNQ channels from heterologous expression studies (0.7 μM for ICA 69673 and 0.4 μM for retigabine, both assayed with KCNQ2/3 heteromeric channels) (Figures 2A,B, 3A, left). This similarity of action by two activators which act on different portions of the channel subunit, and the congruence of the dose-response profiles for *in vitro* respiratory rhythm depression and activation of recombinant KCNQ channels, provides strong pharmacological evidence for involvement of KCNQ potassium channels in the modulation or generation of preBötC inspiratory rhythms.

### DAMGO-Suppressed Inspiratory Rhythms Are Reversed by KCNQ Blockers, but Not by a GIRK-Specific Blocker

MOR1-mediated activation of GIRK potassium channels within the preBötC inspiratory circuit is the prevailing hypothesis offered for the mechanism for OIRD *in vivo* (Montandon et al., 2011, 2016a,b; Montandon and Horner, 2014b; but see Lalley et al., 2014; Montandon and Horner, 2014a; Stucke et al., 2015). We tested this mechanism by bath applying the GIRK-specific peptide blocker TertiapinQ (TPQ) (Jin et al., 1999) onto preBötC slices, after complete suppression of inspiratory rhythmic activity by 100 nM DAMGO. Contrary to expectations, we observed no reversal of DAMGO-mediated inspiratory suppression by blocking GIRK channels, even at high concentrations of TPQ (100 nM) sufficient to block most neuronal GIRK channels (GIRK1/4, IC_50s_ = 15 nM; GIRK1/2, IC_50s_ = 5.4 nM; GIRK2, IC_50s_ = 7 nM) (Jin and Lu, 1999; Jin et al., 1999; Kubo et al., 2005, Li D. et al., 2016) (Figures 2E, 3B, right). Slight increases in integrated baseline activity were reliably observed after TPQ application suggesting that diffusion of the peptide within the slice was not a limiting factor, however sustained organized bursts were never observed. Furthermore, direct application of the GIRK activator ML297 failed to completely mimic DAMGO-mediated *in vitro* OIRD even at concentrations ∼10–100-fold greater than the EC50s for GIRK activation (GIRK1/2, EC_50_ = 0.16 μM; GIRK1/4, EC_50_ = 0.89 μM; GIRK1/3, EC_50_ = 0.91 μM) (Kaufmann et al., 2013; Figures 2F, 3A, right). We concluded from these pharmacological studies that MOR1-mediated activation of GIRK potassium channels does not contribute significantly to suppression of preBötC inspiratory circuits, *in vitro*.

We next tested the role for KCNQ potassium channels in MOR1-mediated depression of preBötC inspiratory rhythms. Depression of inspiratory rhythms by activated KCNQ channels could occur either through a general suppression of neural excitability, or KCNQ channels could serve a more specific modulatory role in OIRD of the preBötC inspiratory circuit. To test these possibilities, KCNQ-specific blockers were applied to preBötC slices after suppression of inspiratory rhythmic activity with DAMGO (100 nM). We reasoned that recovery of inspiratory rhythms in the presence of DAMGO suppression would provide evidence for a specific role of KCNQ channels in the inspiratory network.

**FIGURE 4:**
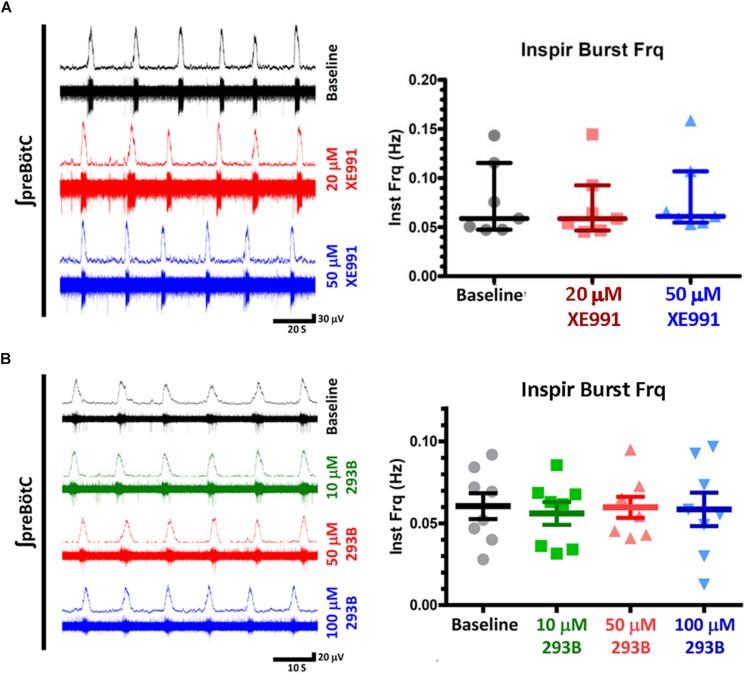
KCNQ blockers (XE991, Chromanol 293) have no effect on baseline inspiratory frequencies from *in vitro* preBötC transverse slice preparations. **(A)** Examples of integrated extracellular preBotC recordings with corresponding voltage traces (left) and summary plots (right) for XE991. **(B)** Integrated extracellular preBotC recordings with corresponding voltage traces (left) and summary plots (right) for Chromanol 293B (293B). No statistical significance observed between baseline and all measured concentrations, for XE991 (*N* = 7) or Chromanol 293B (*N* = 8). Median and IQR plotted for XE991; mean and SE plotted for Chromanol 293B, with individual replicant values.

Two KCNQ open channel blockers, XE991 and Chromanol 293B, were applied after suppression of inspiratory bursts by DAMGO. Both KCNQ blockers were found to restore inspiratory burst frequencies in the presence of 100 nM DAMGO, a concentration that completely suppressed inspiratory rhythmic activity (Figures 2C,D, 3B, left). Control application of these blockers alone had no effect on baseline inspiratory frequency (Figure 4). These two KCNQ blockers are known to exhibit different specificities for different molecular species of KCNQ channels formed as homo- and heteromeric tetrameric combinations of the 5 vertebrate KCNQ α-subunits (Jentsch, 2000). Chromanol 293B shows greater specificity for KCNQ1/KCNE1 heteromeric channels found in cardiac myocytes and epithelial cells (IC_50_ = ∼11 μM), than for neuronal KCNQ channels assembled from KCNQ2-4 (IC_50_ > 500 μM). By exception among neuronal KCNQ2-5 channels, only homomeric KCNQ5 channels are blocked by Chromanol 293B, with a ∼fivefold higher specificity (IC_50_ = ∼100 μM) compared to other neuronal KCNQ channels (KCNQ2/3 and KCNQ4) (Lerche et al., 2007). Conversely, XE991 effectively blocks cardiac/epithelial KCNQ1/KCNE1 and all neuronal KCNQ channels with a moderate to high degree of specificity (IC_50_ < 10 μM), with the exception of homomeric KCNQ5 which displays a ∼fivefold decrease in specificity (IC_50_ = ∼50 μM) (Wang et al., 1998, 2000; Lerche et al., 2000; Schroeder et al., 2000, 2001). Dose-response profiles of these blockers indicate that rescue was achieved by Chromanol 293B at concentrations sufficient to block homomeric KCNQ5 channels (50–100 μM), without blocking other neuronal KCNQ2-4 channel subtypes. Conversely, significant rescue with XE991 only occurred with relatively high concentrations (20–50 μM), consistent with block of KCNQ5 channels. No rescue was observed at lower XE991 concentrations which presumably blocked other neuronal KCNQ2-4 channels (IC_50_s < 10 μM). The lack of rescue by XE991 at lower concentrations (<10 μM) suggests a subordinate role for KCNQ2-4 neuronal subtypes in rescue from OIRD. Taken together these results suggest a specific role for KCNQ channels in modulating OIRD in the preBötC inspiratory network, and implicate a dominant role for KCNQ5 channel subtypes in this modulatory process. However, ICA 69673 shows poor specificity for activating KCNQ5/3 heteromeric channels (Padilla et al., 2009; Wang et al., 2017), complicating this interpretation. Modulation of OIRD by KCNQ channels *in vitro* may thus involve a mixture of different KCNQ molecular species.

### KCNQ, GIRK, and MOR1 Transcripts Are Expressed in preBötC, However, KCNQ Is Not Directly Coupled to MOR1 Intracellular Signaling

**FIGURE 5:**
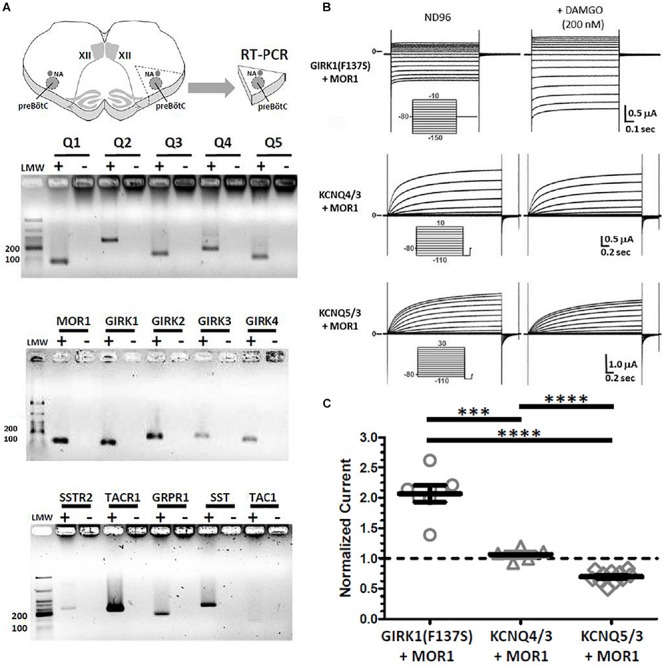
RT-PCRs of micro-dissected preBötC “islands” reveal transcripts for *Kcnq1-5*, *Girk1-4*, and *Mor1*. However, heterologous co-expression of MOR1 with KCNQ4/3 or KCNQ5/3 in *Xenopus* oocytes provide no evidence for direct coupling downstream of activated MOR1. **(A)** Transcripts for all *Kcnq1-5* (Q1–Q5) subunits were detected, along with mu-opioid receptor (*Mor1*) and *Girk1-4* subunits. Known preBötC molecular markers also robustly detected, including Substance P receptor (*Tacr1*), gastrin-release peptide receptor (*Grpr1*), somatostatin (*Sst*). Weaker reactions detected for somatostatin receptor (*Sstr2*), and Substance P (*Tac1*). For each primer set, paired RT-PCRs were performed with first-strand single-stranded cDNAs generated with (+) and without (-) reverse transcriptase. Expected sizes for PCR products (in bp): *Kcnq1* (70), *Kcnq2* (264), *Kcnq3* (117), *Kcnq4* (123), *Kcnq5* (89), *Mor1* (114), *Girk1* (102), *Girk2* (174), *Girk3* (85), *Girk4* (152), *Sstr2* (246), *Tacr1* (221), *Grpr1* (169), *Sst* (250), *Tac1* (110). **(B)** Two-electrode voltage clamp recordings from *Xenopus* oocytes co-injected with cRNAs encoding MOR1 and either GIRK1(F127S), KCNQ4+KCNQ3 (KCNQ4/3) or KCNQ5+KCNQ3 (KCNQ5/3). Current traces are shown before (left) and after (right) bath application of DAMGO (200 nM). Activation of MOR1 by DAMGO augments GIRK1(F127S) currents twofold, but had no effect on KCNQ4/3 or KCNQ5/3 current amplitudes. Reduction of KCNQ5/3 currents reflects time-dependent “rundown,” independent of DAMGO application. **(C)** Summary of current amplitudes after DAMGO (200 nM) for GIRK1(F127S) + MOR1 (*N* = 7), KCNQ4/3 + MOR1 (*N* = 5) and KCNQ5/3 + MOR1 (*N* = 11), normalized to controls without DAMGO. Current amplitudes plotted as median and IQR, with individual replicant values. Statistical significance: ^∗∗∗^*p* = 0.0006, ^∗∗^*p* = 0.0022–0.0025, by Mann–Whitney.

To verify the presence of *Kcnq*, *Girk*, and *Mor1* transcripts in preBötC, RT-PCR was performed on isolated preBötC “islands,” micro-dissected from transverse slices used for *in vitro* recordings (Figure 5A). RT-PCR confirmed the expression of all neuronal *Kcnq* subunits (*Kcnq2-5*) in preBötC, as well as the presence of *Kcnq1* transcripts. *Kcnq1* expression is enriched in cardiac, epithelial and vascular smooth muscle cells (Sanguinetti et al., 1996; Chouabe et al., 1997; Brueggemann et al., 2011; Stott et al., 2013). A positive *Kcnq1* RT-PCR signal from preBötC tissue may thus reflect expression from residual vasculature left in our tissue sample rather than specific neuronal expression, although this possibility is unresolvable at our level of analysis. RT-PCR also confirmed the presence of *Mor1* and *Girk* subunits, particularly *Girk1, Girk2*, and *Girk3* which are known to express widely in neurons of the brain, where GIRK1/2 heteromeric channels are the predominant species (Rifkin et al., 2017). Although *Girk4* expression is known to be predominantly cardiac, limited neuronal expression has been found in the brain, particularly in inferior olive cells (Wickman et al., 1997, 1998). Our positive *Girk4* RT-PCR signal may reflect contamination from neighboring inferior olive cells in our isolated preBötC islands. Nonetheless, RT-PCR showed strong signals for known preBötC molecular markers including, tachykinin receptor (*Tacr1*), somatostatin (*Sst*), and gastrin-releasing peptide receptor (*Grpr1*) related to sighs (Gray et al., 2001, 2010; Li P. et al., 2016). Weaker signals for somatostatin receptor (*Sstr2*) and substance P (*Tac1*) were also observed. Thus, transcripts for *Kcnq* and *Girk* potassium channel subunits, along with the mu-opioid receptor (*Mor1*) were all found to be present in micro-dissected preBötC islands.

*In vivo* KCNQ activation could suppress inspiratory rhythms as molecular effectors coupled to intracellular signaling pathways downstream of MOR1 activation, or through an independent mechanism. Direct coupling to MOR1 as a possibility was suggested by prior studies which reported modulation of native M-currents by somatostatin and dynorphins (Moore et al., 1988; Madamba et al., 1999; Qiu et al., 2008). Although all KCNQ channels are known to couple to Gα_q_-GPCRs, these previous studies suggested coupling between KCNQ channels with Gα_i/o_-and Gα_s_-GPCRs, including somatostatin, opioid, and β-adrenergic receptors, in neurons and smooth muscle cells (Moore et al., 1988; Sims et al., 1988; Madamba et al., 1999; Qiu et al., 2008). Therefore, heterologous expression studies were undertaken to test this possibility using recombinant KCNQ potassium channels and MOR1 coexpressed in *Xenopus* oocytes. Control oocytes injected with cRNAs encoding MOR1 and GIRK1(F137S) carrying a missense mutation in the P-loop which permits functional expression of homomeric channels (Chan et al., 1996), expressed inward rectifying currents that augmented twofold with bath-applied DAMGO (200 nM). However, similar experiments co-expressing MOR1 with either KCNQ5/3 or KCNQ4/3 heteromeric channels showed no effect following bath applied DAMGO (Figures 5B,C). Therefore, we observed no evidence for direct coupling between MOR1 receptors and KCNQ channels from these heterologous reconstitution experiments. Thus, we hypothesized that depression of inspiratory rhythmic activity by activated KCNQ channels likely occurs through a separate mechanism, independent of direct coupling to MOR1 downstream of G-protein signaling.

### Genetic Loss of *Cacna1a* (P/Q-Type) Calcium Channels Increases Sensitivity to OIRD

Suppression of presynaptic voltage-gated calcium channels (P/Q-, N-, and R-type) by Gα_i/o_-coupled GPCRs such as MOR1, via membrane-delimited signaling through Gβ/γ released by ligand binding is a well-established phenomenon (Dunlap and Fischbach, 1978; Herlitze et al., 1996; Ikeda, 1996; Colecraft et al., 2001; Agler et al., 2005; Zamponi and Currie, 2012), although its potential role in OIRD has not been examined, to our knowledge. We took advantage of a mouse KO strain defective for *Cacna1a* (Jun et al., 1999) which encodes the P/Q-type Ca_v_2.1 calcium channel to decrease presynaptic calcium channel function, in order to genetically mimic this effect on OIRD sensitivity. We previously demonstrated that loss of *Cacna1a* function in this strain suppresses sighs and reduces evoked EPSPs in inspiratory interneurons in preBötC slices (Koch et al., 2013).

**FIGURE 6:**
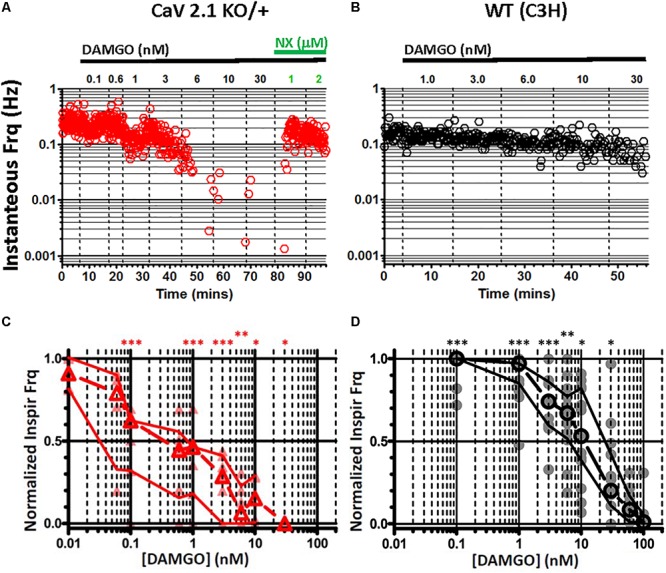
Genetic reduction of Ca_V_2.1 (*Cacna1a*) function sensitizes *in vitro* preBötC inspiratory rhythms to depression by DAMGO. **(A)** Continuous plot of instantaneous burst frequency from a heterozygous Ca_V_2.1 KO/+ preBötC slice, in response to increasing titers of DAMGO (0.1, 0.6, 1, 3, 6, 10, 30, in nM), followed by Naloxone (NX) (1, 2, in mM); dashed lines mark solution exchanges. Steep reduction of burst frequency at 6 nM DAMGO **(B)** WT (C3H strain) preBötC slice instantaneous burst frequency plot, in response to increasing titers of DAMGO (1, 3, 6, 10, 30, in nM); dashed lines mark solution exchanges. Slight depression of burst frequency with 6–10 nM DAMGO. **(C)** Summary of burst frequencies in response to DAMGO titers for heterozygous Ca_V_2.1 KO/+ (*N* = 6) preBötC slices. **(D)** Summary of burst frequencies in response to DAMGO titers for WT (*N* = 16) preBötC slices. Heterozygous Ca_V_2.1 KO/+ respiratory burst frequencies are increased ∼10-fold in sensitivity to DAMGO depression, relative to WT (Ca_V_2.1 KO/+ IC_50_ = ∼1 nM; WT IC_50_ = ∼10 nM). Median and IQR plotted, with individual replicant values. Statistical significance: ^∗∗∗^*p* = 0.0003–0.0009, ^∗∗^*p* = 0.0012, ^∗^*p* = 0.0159–0.0188 by Mann–Whitney.

Sensitivity to OIRD was assessed in preBötC slices obtained from mutant Ca_V_2.1 KO and WT animals, by recording inspiratory burst frequency as a function of increasing concentrations of bath applied DAMGO. As an example, continuous plots of instantaneous inspiratory burst frequency as a function of time and drug application are shown in Figure 6, for heterozygous Ca_v_2.1 KO/+ and WT preBötC slices derived from the same genetic background (C3H). Strikingly, inspiratory burst frequency declined precipitously in heterozygous Ca_V_2.1 KO/+ slices at DAMGO concentrations exceeding 6 nM, and were completely suppressed at 10 nM (Figure 6A). DAMGO suppression was reversed by the addition of naloxone (1 μM), a MOR1-specific competitive antagonist demonstrating reversibility. By contrast, WT (C3H) burst frequency was largely unaffected by DAMGO concentrations at 6–10 nM, with an IC_50_ of ∼10 nM, similar to C57BL/6J (Figure 6B).

Median dose-response profiles for multiple WT (C3H) and heterozygous Ca_V_2.1 KO/+ slices are shown in Figures 6C,D. Although initial inspiratory burst frequencies were indistinguishable between Ca_V_2.1 KO/+ and WT slices, the loss of one copy of *Cacna1a* in heterozygous slices resulted in a ∼10-fold greater sensitivity to suppression by DAMGO (Ca_V_2.1 KO/+, IC_50_ = ∼1 nM; WT, IC_50_ = ∼10 nM). Inspiratory bursts from homozygous Ca_V_2.1 KO/KO slices were less frequent and more irregular than heterozygous KO/+ or WT slices (Koch et al., 2013), and thus precluded quantitative analysis.

Taken together, these results are consistent with a major role for presynaptic voltage-gated calcium channels in OIRD. We hypothesized that compromised synaptic transmission due to acute MOR1-mediated suppression of presynaptic calcium channels may serve as a major mechanistic component underlying OIRD in the preBötC.

### mEPSC Recordings From *Dbx1*^+^ Inspiratory Interneurons Support a Presynaptic Site of Action for OIRD and Modulation by KCNQ K^+^ Channels

**FIGURE 7:**
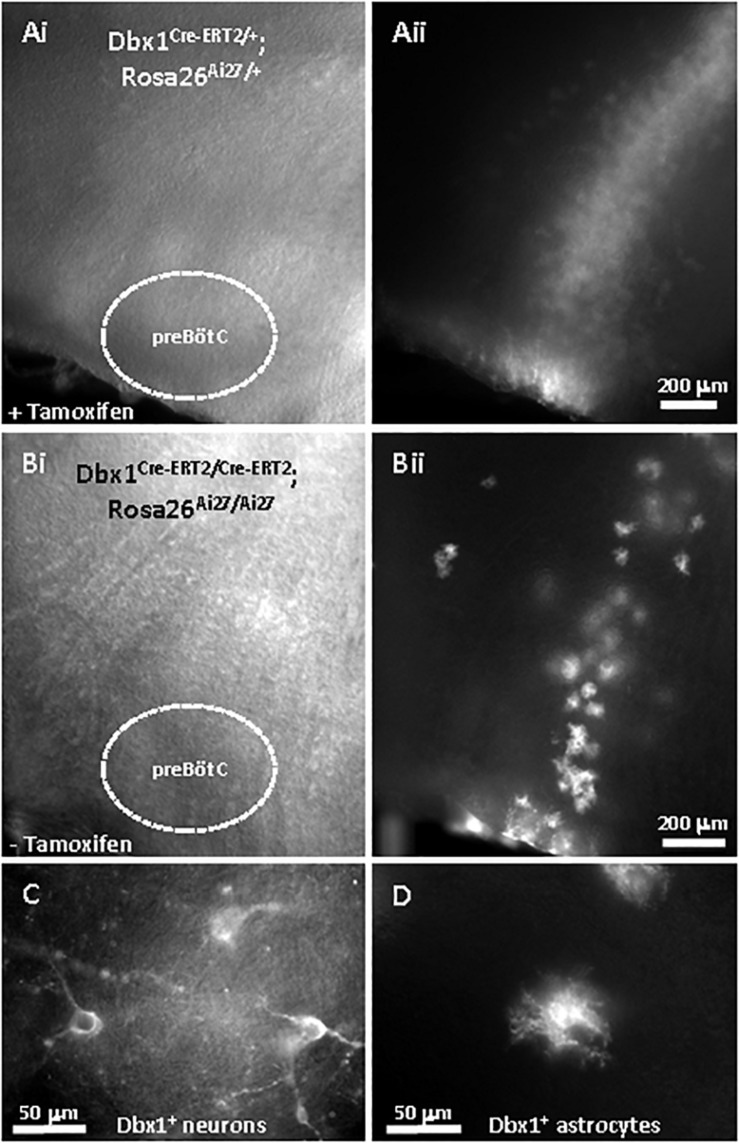
Sparse labeling of *Dbx1*-derived (*Dbx1*^+^) preBötC neurons using *Dbx1*^*Cre–ET2*^ mouse line, without tamoxifen treatment. **(A)** Embryonic tamoxifen injection (e10.5) labels a dense column of *Dbx1*^+^ cells with tdTomato extending radially from the dorsal ventricular surface through the ventrally-located preBötC, viewed by Dodt-IR optics **(Ai)** and RFP fluorescence **(Aii)**. This slice from a P7 animal heterozygous for *Dbx1*^*Cre–ERT2*^ and Ai27 (*Dbx1^*Cre–ERT2/*+^*; *Rosa26^*Ai27/*+^*). **(B)** Similar slice without tamoxifen sparsely labels individual *Dbx1*^+^ cells in a similar pattern as in 7A. This slice from an P7 animal homozygous for *Dbx1*^*Cre–ERT2*^ and Ai27 (*Dbx1*^*Cre–ERT2/Cre–ERT2*^; *Rosa26*^*Ai27/Ai27*^). Viewed by Dodt-IR **(Bi)** and RFP fluorescence **(Bii)**. **(C)** Sparsely-labeled fluorescent neurons and astrocytes **(D)** from the same slice in 7B at higher magnification.

To obtain evidence for a presynaptic site of action for OIRD and KCNQ channels, miniature excitatory post-synaptic currents (mEPSCs) were recorded from genetically-identified *Dbx1*^+^ inspiratory interneurons (Bouvier et al., 2010; Picardo et al., 2013) in the presence of TTX (1 μM) to block spontaneous action potentials. We took advantage of the fortuitous optical clarity provided by sparse labeling of *Dbx1*^+^ cells in Cre-loxP crosses carrying two copies of *Dbx1-CreERT2* with *Rosa26* CAG-floxed-stop reporters expressing either ZsGreen (Ai6) or tdTomato (Ai14) observed in the absence of tamoxifen injections, usually administered to activate conditionally expressed CreERT2 recombinase (Figure 7). Sparse labeling presumably occurred by “leaky” expression of *Dbx1-CreERT2*, since the overall expression pattern without tamoxifen closely resembled that observed with tamoxifen injections at embryonic day 10.5, but at a much lower density of labeled cells. This allowed us to easily distinguish *Dbx1*^+^ neurons from *Dbx1*^+^ astrocytes by morphology for visually-guided patch clamp recordings (Kottick et al., 2017).

**FIGURE 8:**
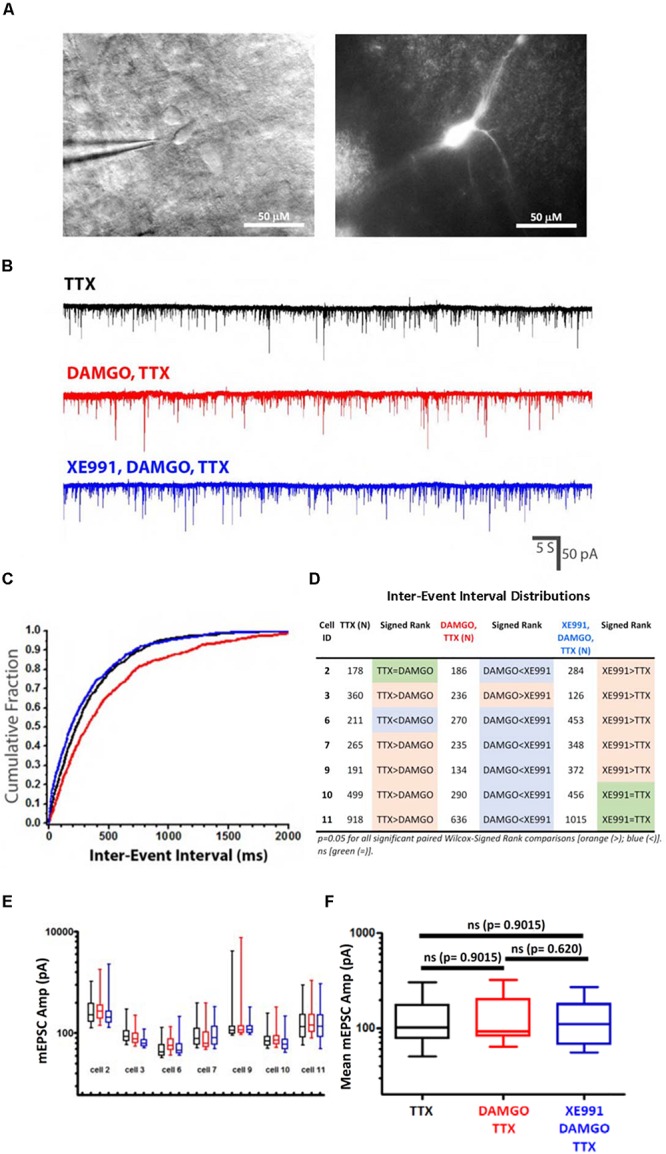
mEPSCs recorded from *Dbx1*-derived (*Dbx1*^+^) preBötC neurons support a presynaptic site of action for DAMGO and KCNQ potassium channels. **(A)** *Dbx1*^+^ preBötC neuron labeled with tdTomato, without tamoxifen, imaged with Dodt-IR (left) and RFP fluorescence (right). Slice homozygous for *Dbx1*^*Cre–ERT2*^ and Ai14 (*Dbx1*^*Cre–ERT2/Cre–ERT2*^; *Rosa26*^*Ai14/Ai14*^). **(B)** Representative mEPSCs recorded from a identified *Dbx1*^+^ inspiratory neuron in response to sequential bath application of TTX (1 μM), DAMGO (100 nM), and XE991 (20 μM). Currents measured at a holding potential of -60 mV. **(C)** Representative cumulative fractional distribution plot of mEPSC inter-event intervals (IEIs) recorded from an inspiratory *Dbx1*^+^ neuron, in response to TTX (black), DAMGO,TTX (red), and XE991,DAMGO,TTX (blue). DAMGO,TTX (red) distribution is significantly shifted toward longer IEIs relative to either TTX (black) or XE991,DAMGO,TTX (blue) distributions; *p* = 0.05 (Paired Wilcox–Signed Rank test; modified Kolmogorov-Smirnov), whereas TTX (black) and XE991,DAMGO,TTX (blue) distributions are not significantly different (Paired Wilcox–Signed Rank test; modified Kolmogorov–Smirnov). **(D)** Summary of pairwise comparisons of mEPSC cumulative fractional inter-event interval distributions (IEIs) from individual neurons in response to TTX (*N* = 7), DAMGO,TTX (*N* = 7) and XE991,DAMGO,TTX (*N* = 7), by paired Wilcox–Signed Rank tests (modified Kolmogorov-Smirnov). Significant ranked differences in mEPSC IEI distributions denoted by colored boxes [*p* = 0.05; orange (>), blue (<)]. Orange denotes statistically significant shifts toward longer IEIs, blue denotes significant shifts toward shorter IEIs. Non-significance denoted by green (=). **(E)** No consistent changes in mEPSC amplitudes from *Dbx1*^+^ neurons in TTX (1 μM) after application of DAMGO (100 nM) and XE991 (20 μM). Mean (bar), SE (box) and minimum/maximum (whiskers) plotted for each recorded neuron following sequential application of TTX (black), DAMGO (red), and XE991 (blue). **(F)** Pooled mEPSC means from each cell (*N* = 7) for each drug treatment (TTX; DAMGO,TTX; XE991,DAMGO,TTX). Pairwise comparisons between all combinations of drug conditions revealed no statistically significant differences (Mann–Whitney).

*Dbx1*^+^ inspiratory interneurons were identified as ZsGreen or tdTomato expressing neurons within the preBötC region which received spontaneous excitatory inspiratory synaptic bursts, synchronous with integrated extracellular population bursts from the contralateral preBötC. TTX (1 μM) selectively blocked large amplitude excitatory synaptic bursts and left a baseline frequency of spontaneous mEPSCs (Figures 8A,B). Addition of DAMGO (100 nM) significantly reduced spontaneous mEPSC frequency (increased inter-event intervals) in 70% (5/7) of recorded *Dbx1*^+^ neurons (Figures 8C,D), without significantly altering the distribution of mEPSC amplitudes (Figures 8E,F). Subsequent addition of XE991 (20 μM) increased mEPSC frequency (increased cumulative accumulation of short inter-event intervals) in 86% (6/7) of recorded neurons (Figures 8C,D). Individual neurons were observed to be responsive to both DAMGO and XE991 (57%, 4/7), only DAMGO (14%, 1/7), or only XE991 (28%, 2/7), indicating heterogeneity and independence of presynaptic modulatory mechanisms at individual synapses. Taken together, these results suggest that suppression of inspiratory rhythm generation in the preBötC primarily occurs through a reduction of synaptic transmission at excitatory glutamatergic synapses by a presynaptic mechanism. Blockade of presynaptic KCNQ channels may sufficiently compensate for MOR1-mediated reduction of synaptic efficacy to restore rhythmic network function.

### *In vivo* OIRD Is Mimicked Strongly by Systemic KCNQ Activator and Weakly by GIRK-Specific Activator, in an Age-Dependent Fashion

To investigate the relevance of our *in vitro* findings to live mice, *in vivo* plethysmography recordings were obtained from unanesthetized neonatal mouse pups (P7–13) and adult mice (P25–P60), by whole-body plethysmography. Plethysmography traces were analyzed by measuring inter-event intervals for a large number of inspirations (>500), excluding segments containing movement artifacts. Mean respiratory frequencies were derived by fitting individual datasets to single Gaussian distributions.

**FIGURE 9:**
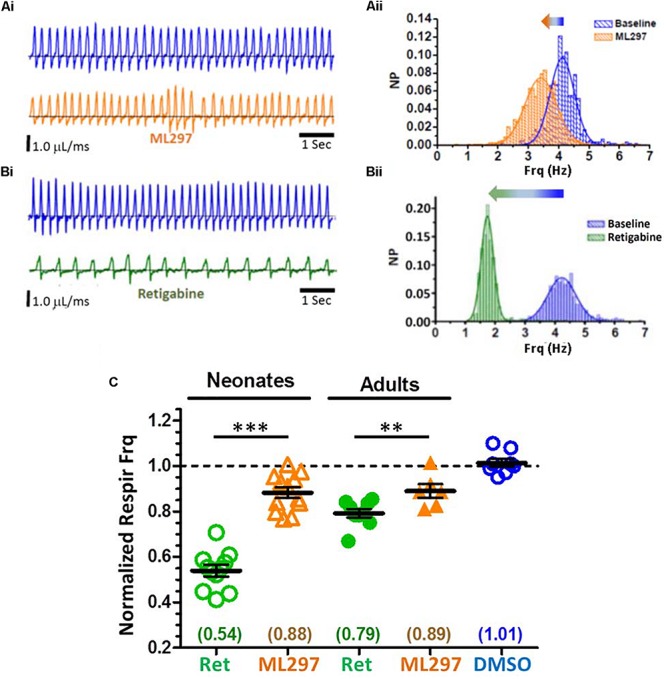
Whole-body plethysmography from awake, unanesthetized neonatal and adult mice, in response to systemic delivery of GIRK1 activator (ML297) or KCNQ activator (retigabine; RTG). Profound suppression of respiratory frequency by KCNQ activator, but only modest suppression by GIRK1 activator. **(A)** Modest reduction of respiratory frequency with ML297 (50 mg/kg, IP) from a neonatal pup. Representative plethysmography recordings before (**Ai**, top), and after drug injection (**Ai**, bottom). **(Aii)** Distributions of instantaneous breath frequencies from the same animal, fitted to single Gaussian distributions. **(B)** Large reduction of respiratory frequency with retigabine (10 mg/kg, IP) from a neonatal pup. Plethysmography records before (**Bi**, top) and after drug injection (**Bi**, bottom). **(Bii)** Distributions of instantaneous breath frequencies from the same animal, fitted to single Gaussian distributions. **(C)** Summary of plethysmography data for neonates (P7–13) and adults (P25–60), normalized to baseline respiratory frequencies. Both neonates and adults exhibited modest and similar reductions (∼10%) in respiratory frequency with ML297. However, retigabine caused large reductions in respiratory frequency in both neonates (46% reduction) and adults (20% reduction). Adult respiratory suppression by retigabine was significantly stronger than ML297 (*p* = 0.012; *t*-test). Results from vehicle controls (DMSO; IP) with neonates and adults were not significantly different and pooled. Population mean and SE plotted, along with individual responses derived from means of fitted Gaussian curves, for each condition, for neonatal retigabine (*N* = 11), neonatal ML297 (*N* = 12), adult retigabine (*N* = 9), adult ML297 (*N* = 6), DMSO control (*N* = 8). Statistical significance: ^∗∗∗^*p* < 0.0001, ^∗∗^*p* = 0.0003, by un-paired *t*–test.

The effect on respiration by systemic activation of GIRK potassium channels was tested after intraperitoneal (IP) injection of ML297 (Figures 9Ai,ii), a small molecular weight activator of heteromeric GIRK1 channels (GIRK1/2, GIRK1/3, GIRK1/4) (Kaufmann et al., 2013; Wydeven et al., 2014). A relatively high dose of ML297 (50 mg/kg) was chosen because IP injections at this dose exert overt behavioral effects in adult mice, including decreased spontaneous locomotion and increased anxiolytic behavior, which provided an independent indicator of drug access into the CNS (Wydeven et al., 2014). ML297 rapidly caused decreased spontaneous locomotion in both pups and adults. In pups, this was followed by the appearance of repetitive whole-body twitches which could be recorded as large amplitude transient pressure artifacts in plethysmography recordings. Despite these gross behavioral changes, respiratory frequency was only marginally decreased after ML297 injection (for pups from 4.5 to 3.9 Hz; for adults 3.1–2.8 Hz). Thus, systemic activation of GIRK potassium channels by ML297 suppressed respiratory frequency only modestly (∼10%) in both neonatal pups and adults, and seems unlikely to contribute significantly to the mechanism of OIRD in unanesthetized mice which can approach a 50% reduction of respiratory rate with morphine at the doses we examined (10 mg/kg for neonates; 150 mg/kg for adults).

We next examined the effect of systemic retigabine, an FDA-approved specific activator of KCNQ potassium channels (Amabile and Vasudevan, 2013; Figures 9Bi,ii). A moderate dose of retigabine was chosen (10 mg/kg), reported to control seizures and prevent tinnitus in rodent models (Li et al., 2013; Kalappa et al., 2015; Ihara et al., 2016). Similar to ML297, IP injections of retigabine rapidly decreased spontaneous locomotion and in pups induced repetitive whole-body twitches. However, unlike ML297, respiratory rates were profoundly suppressed by retigabine in neonatal pups, and significantly reduced in adults (Figure 9C). In neonatal pups, mean baseline respiratory rate of 3.9 Hz was reduced by ∼50% to 2.2 Hz after retigabine injection compared to vehicle controls (*p* = 4.5 e-8; *t*-test). In adults, retigabine reduced respiratory rate by ∼20%, approximately double the reduction observed with ML297 (*p* = 0.012; *t*-test). These results indicate a dominant role for KCNQ potassium channels in the control of *in vivo* eupnic basal respiratory frequency under the unanesthetized state. Systemic activation of KCNQ channels by retigabine significantly depressed respiratory frequency, particularly in neonatal pups (54% of baseline frequency) equal to the depressive effect of systemic morphine (56% of baseline frequency). Importantly, these results suggest that depression of respiratory drive may potentially be an unrecognized adverse side-effect of retigabine at moderate doses, particularly at early ages.

### State-Dependent Reversal of *in vivo* OIRD by Systemic KCNQ Blocker

**FIGURE 10:**
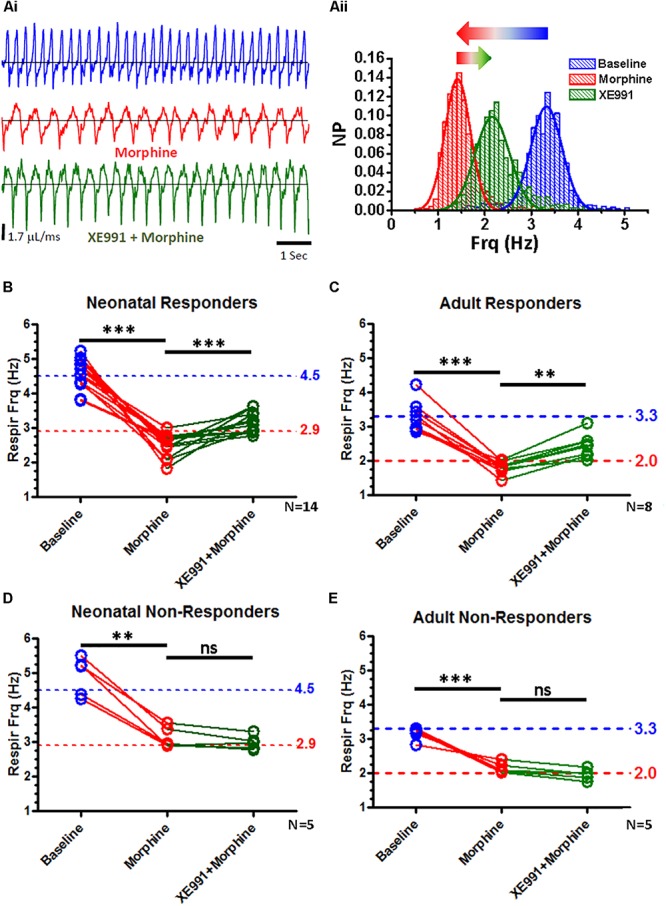
State-dependent reversal of respiratory suppression by morphine with XE991. Whole-body plethysmography from unanesthetized neonatal and adult mice, in response to systemic IP injections with morphine and XE991, a KCNQ-specific blocker. **(A)** Representative plethysmography recordings from an adult “responder” mouse during baseline breathing (**Ai**, blue), after morphine injection (150 mg/kg) (**Ai**, red), and subsequent injection with XE991 (3 mg/kg) (**Ai**, green). **(Aii)** Distributions of instantaneous breath frequencies from the same animal under each condition, fitted to single Gaussian distributions. XE991 rescue of respiratory suppression by morphine was variable, and dependent upon the respiratory frequency observed after morphine injection. **(B)** Neonatal “responders” exhibited strong suppression of respiratory frequency to morphine, below a 2.9 Hz “threshold” frequency. All animals partially recovered respiratory frequency with XE991 (*N* = 14, *p* < 0.0001; paired *t*-test). **(C)** Adult “responders” similarly exhibited strong respiratory suppression to morphine, below a 2.0 Hz “threshold” frequency. All animals partially recovered respiratory frequency with XE991 (*N* = 8, *p* = 0.0003; paired *t*-test). **(D)** Neonatal “non-responders,” by contrast failed to exhibit morphine suppression of respiratory frequency below a 2.9 Hz “threshold.” None of these animals increased respiratory frequency more XE991 (*N* = 5, ns; paired *t*-test). **(E)** Adult “non-responders” similarly failed to exhibit morphine suppression of respiratory frequency below a 2.0 Hz “threshold.” None of these animals responded to XE991 with an increased respiratory rate (*N* = 5, ns; paired *t*-test). ^∗∗∗^*p* < 0.0001, ^∗∗^*p* = 0.0003, ns, non-significant.

We then examined the ability of systemic XE991 to reverse OIRD due to morphine delivered IP, in both neonatal and adult animals. In neonatal animals, morphine injected at 10 mg/kg resulted in a 36% suppression of mean respiratory frequency from 4.5 to 2.9 Hz (Figure 10), but with considerable variance among individual animals. Higher doses of morphine in neonates induced spontaneous locomotion as previously described (Jacquet et al., 1976; Koek et al., 2012), which precluded plethysmography measurements under unrestrained conditions. Adults exhibited a lower mean basal respiratory rate (3.3 Hz), and a dose of 150 mg/kg morphine achieved a similar degree of acute respiratory suppression as neonates (39% reduction of mean respiratory rate).

Responses to subsequent injection with XE991 (3 mg/kg) were found to be dependent upon the breathing rate measured after depression by morphine (Figures 10B–E). When morphine depressed respiratory frequency below a “threshold” frequency (2.9 Hz in neonates and 2.0 Hz in adults), subsequent XE991 increased and partially restored respiratory frequency (from a mean of 2.5 to 3.1 Hz in neonates, a 24% increase; from 1.8 to 2.4 Hz in adults, a 33% increase). By contrast, when morphine failed to depress respiratory rate below these “threshold” frequencies in either neonates or adults, subsequent XE991 failed to increase respiratory rate. We designated these two populations “responders” and “non-responders,” defined by the ability (“responders”) or failure (“non-responders”) to increase respiratory rate depressed under morphine by more than 5%, after subsequent injection with XE991. These two populations exhibited statistically distinct response characteristics, comparing morphine-depressed respiratory rates between “responder” and “non-responder” populations (*p* = 0.025 for neonates, *p* = 0.0043 for adults; Mann-Whitney). Similar non-parametric comparisons between “responder” and “non-responder” populations for baseline and morphine+XE991 respiratory rates were statistically insignificant, with the exception of a significant difference between adult morphine+XE991 responder and adult non-responder populations (*p* = 0.0063; Mann-Whitney). These results suggest a “state-dependence” in the ability of XE991 to restore respiratory rate, dependent upon the degree of individual responsiveness to respiratory depression by morphine. These results are reminiscent of other studies which suggest that the modulatory effects of respiratory neuromodulators are dependent on baseline respiratory frequency (Doi and Ramirez, 2010), and the modulatory state of the animal (Langer et al., 1985). The ability of XE991 to reverse OIRD may thus be similarly dependent upon the internal modulatory state of the animal, defined by basal levels of endogenous respiratory-related neuromodulators (Langer et al., 1985; Gray et al., 1999; Doi and Ramirez, 2010; Li P. et al., 2016; Yackle et al., 2017).

## Discussion

**FIGURE 11:**
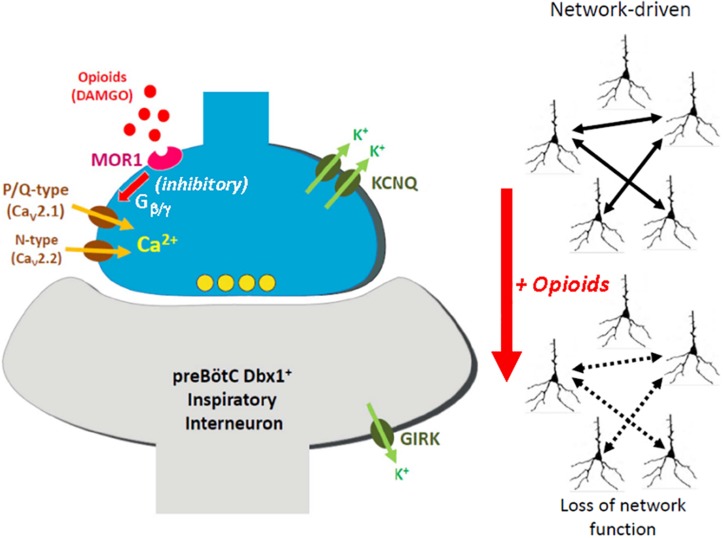
Model for presynaptic site of action by opioids underlying opioid-induced respiratory depression (OIRD). We hypothesize that glutamatergic presynaptic terminals onto *Dbx1*-derived (*Dbx1*^+^) inspiratory interneurons within the preBötC compartmentalize mu-opioid receptors (MOR1) and KCNQ potassium channels, in addition to the normal complement of presynaptic calcium channels associated with vesicular release, including P/Q-type (Ca_V_2.1) and N-type (Ca_V_2.2) calcium channels. Activation of MOR1 leads to suppression of presynaptic calcium channel function, via direct binding with Gβ/γ generated by receptor activation. This leads to reduced synaptic transmission across the excitatory inspiratory circuit, defined by an essential kernel of *Dbx1*^+^ glutamatergic neurons. Because rhythmogenesis within this circuit is largely network-driven, reduced synaptic efficiency leads to collapse of rhythmogenic capacity, and opioid-induced apnea. We further propose that active KCNQ potassium channels contribute to maintaining presynaptic resting membrane potentials. Blocking these channels may depolarize presynaptic terminals, mitigating the effects of opioid-mediated suppression of calcium channel function. We emphasize that our model proposes that presynaptic KCNQ channels act independent of direct signaling downstream from MOR1 activation, but function in parallel with MOR1-mediated inhibition of presynaptic calcium channels by virtue of co-compartmentalization to the presynaptic terminal. Sufficient restoration of synaptic transmission may thus revive rhythmogenesis, in spite of ongoing signaling by activated MOR1. Our model of OIRD suggests a relatively minor role for MOR1-coupled activation of GIRK potassium channels, however somatic GIRK currents have been reported by other studies.

Our study suggests that opioids act presynaptically and are modulated by presynaptic KCNQ channels. As shown in Figure 11, we hypothesize that presynaptic terminals which provide excitatory input for the essential kernel of glutamatergic respiratory interneurons, contain both MOR1 opioid receptors and KCNQ potassium channels, in addition to the normal complement of presynaptic calcium channel (P/Q-, N-, and/or R-type) and vesicular release molecular machinery. Opioids are known to activate presynaptic MOR1 at many synapses (Vaughan et al., 1997; Tao and Auerbach, 2005; Zhu and Pan, 2005; Hjelmstad et al., 2013), which signal to reduce voltage-dependent calcium influx by a well-described Gα_i/o_ signaling pathway. Downstream of receptor activation, presynaptic calcium channels are inhibited by membrane-delimited binding between Gβ/γ released from activated MOR1, and the second intracellular loop between homology domains I and II of P/Q- and N-type calcium channels (Herlitze et al., 1996; Ikeda, 1996; Zamponi et al., 1997; Agler et al., 2005). Although our results demonstrate a specific role for P/Q-type calcium channels in preBötC rhythm generation consistent with MOR1-mediated OIRD, a similar role for N-type calcium channels likely operates within the preBötC (Lieske and Ramirez, 2006; Koch et al., 2013).

Suppression of calcium channel function occurs in part by slowing activation kinetics and shifting intrinsic voltage-dependence toward depolarized potentials, thus compromising evoked synaptic transmission (Zamponi and Currie, 2012). Based on our data, we hypothesize that independently, partially activated presynaptic KCNQ potassium channels contribute to setting basal presynaptic membrane potential, as observed in hippocampal culture and slices (Vervaeke et al., 2006; Peretz et al., 2007) and the glutamatergic Calyx of Held (Huang and Trussell, 2011). Thus, blockade of KCNQ channels with XE991 may depolarize presynaptic terminals sufficiently to compensate for the reduced function of calcium channels inhibited by bound Gβ/γ. The inhibitory action of Gβ/γ on calcium channels is voltage-dependent, and reversible by strong depolarizations (Colecraft et al., 2000; Zamponi and Currie, 2012). However, how much calcium channel activity may be restored by the likely modest depolarization resulting from blockade of presynaptic KCNQ channels is unclear, and other mechanisms may explain our results. Direct measurements of Ca^2+^ influx by genetic approaches expressing genetically-encoded calcium sensors may provide a direct test of this hypothesis (Dreosti et al., 2009; Hoppa et al., 2014).

This model for OIRD is consistent with an essential role for glutamatergic transmission in preBötC rhythmogenesis (Funk et al., 1997; Del Negro et al., 2010), and a determinative influence of glutamatergic synaptic dynamics in shaping network rhythmicity (Guerrier et al., 2015; Kottick and Del Negro, 2015). Our findings are also consistent with previous demonstrations that ampakines, which act as enhancers of glutamatergic transmission, effectively rescue OIRD and other forms of apnea (Ren et al., 2006, 2009, 2015; Lorier et al., 2010; Ogier et al., 2007). 5HT4a receptor agonists (Manzke et al., 2003) and other treatments that elevate intracellular cAMP (Ballanyi et al., 1997; Ruangkittisakul and Ballanyi, 2010) have also been reported to rescue OIRD, suggesting additional mechanisms mediated by PKA. We demonstrate that excitatory input onto *Dbx1*-derived neurons are presynaptically inhibited by DAMGO. There is growing evidence that *Dbx1*-derived neurons are necessary and sufficient for respiratory rhythmogenesis (Hayes et al., 2012; Wang et al., 2014), suggesting that these depressive effects in preBötC may contribute to OIRD. However, we cannot exclude the possibility that DAMGO may also critically affect other neurons within the brainstem respiratory network.

An additional caveat is that our *in vitro* slice studies were conducted in ACSF elevated from 3 to 8 mM, experimentally necessitated to evoke consistent inspiratory rhythms. *In vitro* transverse preBotC slices typically fail to generate reliable respiratory bursts in 3 mM K^+^. Raising external potassium from 3 to 8 mM would be expected to shift the potassium reversal potential from -98.5 to -73.8 mV, assuming an internal K^+^ concentration of 150 mM, based on a Nernst equilibrium potential. For non-voltage gated potassium channels such as GIRK, this might be expected to reduce its relative repolarization influence compared to voltage-gated KCNQ potassium channels, due to a combination of reduced K^+^ driving force, and fixed intrinsic outward conductance for GIRK channels versus non-linear voltage-dependent increases in conductance for KCNQ channels. Other determinative factors include the relative expression levels of these channel types. Whether these considerations may significantly alter the relative roles of these two potassium channels on burst generation in preBotC circuits may require computational studies in realistic network models, with biophysically accurate GIRK and KCNQ conductances, along with further experimental studies. However, the combination and concordance of our *in vitro* and *in vivo* experimental results suggests a relatively minor role for GIRK activation compared to KCNQ activation in suppressing respiratory bursts.

Alternative scenarios could also contribute. Gβ/γ suppression of synaptic transmission is described at some synapses, which bypasses direct involvement of calcium channels or altered influx of Ca^2+^ (Blackmer et al., 2001, 2005; Gerachshenko et al., 2005). By this mechanism, Gβ/γ interferes with the SNAREs that comprise the vesicular release mechanism, by binding to SNAP-25, one of the three key proteins which forms the ternary SNARE complex. This prevents Ca^2+^-induced vesicular fusion, perhaps by sterically interfering with synaptotagmin/Ca^2+^ action (Zurawski et al., 2017). Another possible mechanism is based on the recently revealed voltage-dependence of many GPCRs, including MOR1. Many GPCRs respond to membrane depolarization with reduced affinity for agonist binding, and a gating current indicative of an intrinsic voltage sensor (Ben-Chaim et al., 2006; Ohana et al., 2006; Parnas and Parnas, 2007). The basis for this voltage-dependence derives from a Na^+^ ion that occupies a site within the ligand binding pocket, which is required for high affinity ligand binding (Livingston and Traynor, 2014; Vickery et al., 2016). Depolarization electrostatically displaces Na^+^ from this pocket and lowers ligand affinity. Conceivably, depolarizations caused by blocking KCNQ channels may reduce the ligand binding affinity of presynaptic MOR1 directly at the presynaptic terminal, or by promoting repetitive depolarizations in intrinsic burster neurons (Ramirez et al., 2011, 2012), sufficiently to decrease MOR1 signaling. Whether these possible mechanisms operate within preBötC respiratory neurons to contribute to OIRD await future studies.

Rescue of *in vivo* OIRD by KCNQ blockers was more variable than that observed with *in vitro* slices. Several reasons may explain increased *in vivo* variability, an issue of clinical importance since this variability makes OIRD potentially unpredictable and dangerous. First, opioids may act at multiple brainstem sites affecting respiration (Stucke et al., 2008, 2015), including the Kolliker-Fuse nucleus which provides excitatory drive onto the respiratory network, counteracting apneusis (Molkov et al., 2013; Smith et al., 2013; Levitt et al., 2015). Inhibition of pontine structures exaggerates OIRD (Stucke et al., 2008, 2015; Prkic et al., 2012). Secondly, glutamatergic transmission within the preBötC is affected by multiple convergent neuromodulators, including norepinephrine, serotonin and substance P (Doi and Ramirez, 2008; Funk et al., 2011; Ramirez et al., 2012). Conceivably, multiple Gα_q_-coupled neuromodulators may act on the same pool of presynaptic terminals via decreased PIP2 levels to regulate basal transmitter release by modulating KCNQ potassium channels (Suh and Hille, 2002; Hoshi et al., 2003; Suh et al., 2006; Sun and MacKinnon, 2017) and other presynaptic effectors. Thirdly, opioids may lead to compensatory release of other neuromodulators (Muere et al., 2014; Langer et al., 2017), or disinhibition of excitatory circuits, leading paradoxically to increased agitation and hyperactivity (Hnasko et al., 2005; Mustapic et al., 2009; Koganezawa et al., 2011). In addition, differing degrees of systemic hypoxia between “responder” and “non-responder” individuals may contribute to state-dependence of recovery by KCNQ blockers. All these considerations may interact to result in *in vivo* OIRD state-dependence, and variable efficacy of pharmacological interventions.

In contrast to our findings, OIRD has been proposed to be caused by MOR1-mediated post-synaptic activation of GIRK potassium channels (Montandon et al., 2016a, b). Moreover, previous *in vitro* studies have reported upregulation of a potassium current (Gray et al., 1999) and small hyperpolarizing shifts in basal membrane potentials in inspiratory preBötC neurons (Montandon et al., 2011) in response to MOR1 activation. However, the upregulated potassium current reported in Gray et al. (1999) exhibited outward rectification, rather than inward rectification which would be expected for a GIRK conductance (Rifkin et al., 2017). Reports of baseline hyperpolarization by opioids are also limited and inconsistent between different laboratories (Takeda et al., 2001; Ballanyi et al., 2009, 2010; Montandon et al., 2011). In balance, in our judgment these studies provide inconclusive evidence for widespread MOR1 activation of post-synaptic GIRK channels, despite our results which show the presence of *Girk* transcripts in the preBötC. What elements of this model of GIRK-mediated suppression of excitability may contribute to OIRD remains an open question.

Our studies support a modest role for GIRK channels in OIRD, either by the failure to rescue *in vitro* slice models of OIRD with a GIRK-specific blocker (TertiapinQ), or to mimic OIRD by systemic delivery of a GIRK1-specific activator (ML297) *in vivo*, under unanesthetized conditions. Under these conditions, ML297 likely activates all GIRK1-containing tetrameric channel species, including the predominant GIRK1/2 heteromeric channel species found in brain (Rifkin et al., 2017). However, our pharmacological results cannot exclude the possibility that a component of OIRD may be mediated by homo- or heteromeric GIRK2/GIRK3 channels (GIRK2/3, GIRK2, GIRK3) not activated by ML297, even though these are considered relatively minor GIRK species. Several major experimental and interpretative differences could underlie the discrepancies between our conclusions from those proposed by Montandon et al. (2016a, b). In these studies, drug delivery and *in vivo* experimental measurements were made under anesthesia with 1.5–2.5% isoflurane. Isoflurane suppresses respiratory drive, and even at low concentrations may potentiate the depressive effects of other opioid-related respiratory suppressants. The use of isoflurane thus complicates the interpretation of *in vivo* results. By contrast, urethane is the anesthetic of choice for *in vivo* respiratory studies, since it yields effective analgesia with minimal cardiorespiratory suppression (Doi and Ramirez, 2010; Pagliardini et al., 2012). Alternatively, systemic delivery of drugs without anesthetics as in our study, may provide a more accurate measure of opioid action on *in vivo* respiratory drive. Another significant interpretative error comes from the stated use of flupirtine as a GIRK-specific activator (Montandon et al., 2016b). Flupirtine is an early analog of retigabine. Both are generally recognized as specific activators of KCNQ, not GIRK potassium channels. Despite limited off-target effects including potentiation of δ-subunit containing GABA_A receptors (Klinger et al., 2015; Treven et al., 2015) and block of Kv2.1 potassium channels (Stas et al., 2016), to our knowledge there are no reports of either flupirtine or retigabine activating GIRK channels. However, off-target potentiation of δ-subunit containing GABA_A receptors by the high concentrations of flupirtine used (200–300 μM) in the *in vivo* microperfusion studies of Montandon et al. (2016a, b, 2017) combined with KCNQ activation, rather than specific GIRK activation, may account for the respiratory depression observed in these studies. These studies also report the loss of respiratory depression by both flupirtine and DAMGO, as well as a reduced augmentative effect by the positive neuromodulator GR73632 (an NK-1 receptor agonist) in homozygous *Girk2* KO mice, compared to WT controls (Montandon et al., 2016a, b). Loss of *Girk2* results in seizure-prone mice and the dysregulation of GIRK1 subunits trafficked to the plasma membrane (Signorini et al., 1997; Ma et al., 2001). Conceivably, compensatory mis-regulation of KCNQ and other ion channels or receptors due to chronic overexcitation in constitutive *Girk2* KO mice may result in DAMGO-dependent OIRD effects different from acute pharmacological manipulations in the WT background. We believe these choices in experimental design and potential errors in data interpretation present important challenges to the prevailing model of GIRK-mediated OIRD (Montandon et al., 2016a, b).

Thus, we conclude that opioids act primarily by broadly disrupting the efficacy of excitatory transmission within the preBötC inspiratory network, leading ultimately to the precipitous collapse of the rhythmogenic capacity of the network. This may occur in a manner functionally similar to the physical ablation of neurons from the network (Hayes et al., 2012; Wang et al., 2014). Consistent with our conclusion, manipulations designed to augment opioid-compromised synaptic transmission, for example by either pharmacologically depolarizing presynaptic terminals with specific presynaptic potassium channel blockers, or by allosterically potentiating the open-time kinetics of post-synaptic AMPA receptors with ampakines (Greer and Ren, 2009), may provide a useful framework and strategy for reversing OIRD. Glutamatergic synaptic transmission is the target of a variety of excitatory neuromodulators (Schneggenburger and Forsythe, 2006; Doi and Ramirez, 2008). The inhibitory action of opioids on glutamatergic synapses, acting in concert with endogenous neuromodulators released in a state-dependent manner, may thus influence the efficacy of *in vivo* respiratory depression exerted by opioids. Unraveling the interplay between endogenous neuromodulators and exogenous opioids acting on synaptic transmission between rhythmogenic neurons will be a critical next step for understanding and preventing mortality associated with OIRD.

## Data Availability Statement

The datasets generated for this study are available on request to the corresponding author.

## Ethics Statement

The animal study was reviewed and approved by IACUC at Seattle Children’s Research Institute.

## Author Contributions

AW designed research, performed research, and analyzed data. AW and J-MR wrote the manuscript.

## Conflict of Interest

The authors declare that the research was conducted in the absence of any commercial or financial relationships that could be construed as a potential conflict of interest.
